# *In Vitro*, *Ex Vivo*, Instrumental, and Clinical Assessment of a Novel Anti‐aging Serum Targeting Oxidative Stress

**DOI:** 10.1111/jocd.16664

**Published:** 2025-04-03

**Authors:** Sayantani Goswami, Jin Namkoong, Marwa El Hajoui, Ewelina Lesniak, Joanna Wu

**Affiliations:** ^1^ Skin Research and Innovation, Global Personal Care and Skin Health R&D Colgate‐Palmolive Company Piscataway New Jersey USA; ^2^ Laboratoires Filorga Paris France; ^3^ Personal Care Product Development, Skin Health R&D Colgate‐Palmolive Company Piscataway New Jersey USA

**Keywords:** aging, cosmetic, hydration, oxidative stress, skin

## Abstract

**Background:**

Primarily driven by oxidative stress, aging results from the attrition of cells, aggravated by environmental stressors. Therefore, protection from oxidative stress is the main target of antiaging cosmetics.

**Aims:**

To evaluate the efficacy of a unique cosmetic serum combining five antioxidants and hyaluronic acid.

**Methods:**

The inactivation of reactive oxygen species by the serum was evaluated *in‐tubo*. IL‐1α release was evaluated using EpiDerm^TM^ skin models, while gene expression analysis and elastin fiber length were evaluated on human skin explants. Finally, the effect of twice daily serum application for 28 days was compared to those induced by a control serum, focusing on instrumental and assessor evaluations.

**Results:**

*In‐tubo*, the serum reduces reactive oxygen species by 45.2%. A single topical application on EpiDerm^TM^ skin models limits UV‐induced ROS‐mediated IL‐1α release. Compared to untreated explants, HB‐EGF (heparin‐binding epidermal growth factor) skin homeostasis marker expression increases by 22‐fold with treatment. Additionally, the serum increases elastin fiber length by 40.2%. Clinically, twice daily application of the serum over a period of 7 days revealed significant improvements in clinical scoring of skin's wrinkle (−12.8%), smoothness (+12.5%), and radiance (+22.2%). The serum also leads to a rapid and long‐lasting increase in skin hydration (30 min: +50.5%, 28 days: +19.9%) and reduced transepidermal water loss (30 min: −7.7%, 28 days: −8.7%). The serum is highly efficacious and well tolerated by the subjects.

**Conclusion:**

The serum has antioxidant, soothing, photoprotective, and moisturizing properties that can be explained by the individual properties of its unique blend of actives.

## 1 Introduction

Facial skin aging is characterized by wrinkles, reduced elasticity, elastosis, irregular pigmentation, fragility, impaired barrier function, dryness, slower healing, and increased vulnerability [1]. These signs result from the combination of intrinsic and extrinsic aging [2]. Intrinsic aging is the natural and genetically programmed senescence of cells, while extrinsic aging is a consequence of the continuous exposure to environmental stressors, UV radiation, and pollutants. Despite some differences, both processes impact identical targets through the generation of reactive oxygen species (ROS). ROS are often considered to play a crucial role in the exacerbation of aging and inflammation [3].

ROS includes superoxide anion (O_2_•‐), hydroxyl radical (OH•), singlet oxygen (^1^O_2_), and hydrogen peroxide (H_2_O_2_), which are by‐products of the normal functioning of the mitochondrial respiratory chain. Under normal conditions, ROS are neutralized by non‐enzymatic antioxidants, damage‐eliminating systems, and detoxifying enzymes [4]. Yet, age and environmental stressors can overwhelm these defense systems, causing oxidative imbalance. The resulting damage to DNA, and the subsequent loss of cellular functions, have long been considered major factors in leading to oxidative stress [5]. Oxidative stress damages all macromolecules, leading to oxidized compounds and further ROS accumulation [6]. Irrespective of the primary cause, ROS generation in excess has multiple deleterious effects on the skin, which include impairing skin barrier function, and weakening the extracellular matrix, leading to increased collagen degradation by matrix metallopeptidase (MMP) [6]. Oxidative stress also increases tyrosine levels, the initial substrate of melanin synthesis, and stimulates melanogenesis, thus enhancing pigmentation defects [7]. Finally, there is increasing evidence that ROS acts as a signaling molecule in senescence induction [8].

Antioxidants, which neutralize ROS and protect cells against oxidative stress, are core active components of anti‐aging cosmetics. Yet, the wide range of available antioxidant compounds presents variable in vivo activities [9]. Some of these compounds have low stability in water‐based emulsions, while others show low lipid solubility and poor capacity to penetrate the skin.

A study of anti‐aging cosmetics sold by international brands revealed that tocopherol and ascorbic acid are the most frequently used antioxidants [10]. Even, if they are generally used in combination with other antioxidants, there is still room for improvement in their combinations. Antioxidant combinations are aimed at (1) protecting against oxidative stress, (2) producing a soothing effect, and (3) enhancing the skin's moisture retention capability, overall mitigating aging signs. Besides restoring the skin barrier, it is essential to prevent the entry of additional pollutants and increase oxidative stress. Having developed a cosmetic serum with the goal of addressing all these challenges, we evaluated its effects in vitro and ex vivo. Additionally, we clinically assessed its efficacy upon repeated applications.

## 2 Materials and Methods

### 2.1 Topical Treatment

The test product is a water‐based cosmetic serum (HYDRA‐AOX, Laboratoires Filorga Cosmétiques, Paris, France). In addition to solubilizers, gel forming compounds, fragrance and preservatives, it includes antioxidants (niacinamide, ascorbyl glucoside, tocopherol, astaxanthin, and ergothioneine), humectants (glycereth‐26 and methylpropanediol), and emollients (pentylene glycol and caprylyl glycol). Its ingredients also include hyaluronic acid and a proprietary complex of compounds inspired by cosmetic injectables.

This test serum was compared to a control serum in which antioxidants, hyaluronic acid, and the proprietary complex were replaced by water. The exact composition of both sera is provided in Supporting Information S1.

### 2.2 ROS Inactivation Assay

The control and test serums' capacity to spontaneously inactivate exogenous ROS production was assessed *in‐tubo* using an adapted version of the ROS/Superoxide Detection Assay Kit (#ab139476, Abcam, MA, USA).

Diluted serum (0.02% dilution of either serum in ROS/Superoxide Detection Assay Kit wash buffer) was allowed to react with 0.3% hydrogen peroxide (used as an exogenous ROS inducer) and the fluorescently tagged Oxidative Stress Detection Reagent. After 1 hour of incubation at room temperature, variation in ROS amounts compared to baseline level was quantified using a SpectraMax M5e plate reader (Molecular Devices, CA, USA).

### 2.3 Human Skin Tissue Models

Experiments were performed in quadruplicate on abdominal skin explants from a 53‐year‐old Caucasian female (GenoSkin, MA, USA). These explants were maintained in the recommended media under optimal conditions (37°C, 5% CO_2_) and received daily topical applications of the control or test serum, while untreated samples received no serum. After 7 days, explants were placed in RNAlater solution (Life Technologies, CA, USA) for gene expression analysis or fixed in 4% paraformaldehyde for immunohistochemical analysis.

Evaluation of IL‐1α release was performed using the commercially available EpiDerm^TM^ skin tissue model (MatTek Life Sciences, MA, USA) that consists of an epidermal layer composed of keratinocytes. Once equilibrated (37°C, 5% CO_2_), the control serum or the test serum was applied to the surface of the tissue after UV irradiation (10 J/cm^2^) using a UV chamber (Suntest CPS+, Atlas, IL, USA). Untreated skin tissue models served as negative controls.

### 2.4 IL‐1α Quantification

Twenty‐four hours after serum application and UV irradiation, IL‐1α quantification was performed on culture media using an ELISA kit (R&D Systems, MN, USA) and following the manufacturer's protocols.

### 2.5 Gene Expression Analysis

RNA from homogenized skin explants (Polytron® PT 10–35 GT, Kinematica, Bohemia, NY, USA) were extracted using the RNeasy Fibrous Tissue Mini Kit (Qiagen, MD, USA). After quantification (NanoDrop Spectrophotometer, Thermo Fisher Scientific, MA, USA) and dilution according to the manufacturer's protocols, cDNA synthesis was carried out using the Maxima First Strand cDNA Synthesis kit (Thermo Fisher, MA, USA). Gene expression analysis by RT‐qPCR was performed using TaqMan® gene expression assays and QuantStudio 7 Flex (Thermo Fisher, MA, USA). Glyceraldehyde 3‐phosphate dehydrogenase (GAPDH) housekeeping genes served as references. Target gene expression of the control‐ or test‐treated samples is expressed relative to that of the untreated control.

### 2.6 Immunohistochemical Labeling of Elastin

Fixed skin explant samples were embedded in paraffin and cut into 5‐μm thick sections. Elastin was labeled using a 1:200 dilution of a recombinant rabbit anti‐elastin monoclonal antibody (#ab307150, Abcam, MA, USA), revealed with a goat anti‐rabbit IgG secondary antibody cross‐absorbed to Alexa Fluor 488 (#A‐11008, Thermo Fisher Scientific, MA, USA). DAPI (Vector Laboratories, CA, USA) was used to counterstain nuclei. Elastin fiber length (μm) was measured using the ImageJ software (Version 1.53s, NIH, MD, USA).

### 2.7 Clinical and Instrumental Evaluation

A clinical and instrumental evaluation was carried out to assess the efficacy of the test serum on various signs of aging. Thirty‐three healthy women between the ages of 28 and 47 (mean ± SD = 38.4 ± 5.9‐year‐old) presenting fine lines and poor skin radiance were recruited. After receiving detailed information, all subjects gave their written informed consent. Besides, this noninvasive cosmetic evaluation strictly conformed to the principles of the Declaration of Helsinki and followed Good Clinical Practices. Performed in Italy under the supervision of Colgate‐Palmolive Company (USA), it was approved by the U.S. Investigational Review BoarInvestigational Review BoardInc. under the reference of U.S. IRB2022CP/09.

Short‐term assessments were performed before and after (30 min and 8 h) a single application of 2 mg/cm^2^ of the test serum on the volar side of a randomly selected forearm, with the untreated volar side of the second forearm being used as a control. For longer‐term evaluations (7 and 28 days), subjects self‐applied the test serum twice daily (morning and evening) onto their clean and dry face before proceeding with their cosmetic routine.

The parameters analyzed at all time points (T0, T30 min, 8 h, Days 7 and 28) are:

- Skin hydration using a CM 825 Corneometer (Courage+Khazaka Electronic GmbH, Germany).
- Transepidermal water loss (TEWL) thanks to a TM 300 Tewameter (Courage+Khazaka Electronic GmbH, Germany).

All other parameters were evaluated at baseline (T0) as well as at days 7 and 28. These correspond to:

- Skin profilometry that was analyzed with the Primos CR (Canfield Scientific, NJ, USA), a contactless 3D skin surface scanner. The analysis focused on the Sa parameter, the arithmetic mean of the surface roughness.
- Clinical evaluations of skin wrinkles, smoothness, and radiance. These evaluations were performed by a trained dermatologist using a 0 to 10 numeric scale, with 0 corresponding to very wrinkled, very rough, and very opaque skin, while 10 corresponds to no visible wrinkle, very smooth, and very bright skin.
- A self‐assessment questionnaire enabled evaluation of the perceived product's tolerance and efficacy. Questions were: “The product is well tolerated,” “The skin seems firmer,” “Fine lines/wrinkles seem smoothed,” “The product smoothes skin's texture,” “The complexion looks brightened,” “The complexion seems more even,” “The product gives a visible youthful effect,” “The product corrects the first signs of aging,” “The skin seems plump,” and “The skin is hydrated.” Possible answers were “totally agree,” “mostly agree,” “mostly disagree,” or “totally disagree.” “Totally agree” and “mostly agree” were considered positive scores.

### 2.8 Statistical Analysis

*In‐tubo*, in vitro, and ex vivo experiment results are expressed as mean and standard deviation (SD) and analyzed using Student *t*‐tests.

Clinical and instrumental evaluation results are presented as the mean and the standard error of the mean (SEM). After verifying normal data distribution with Shapiro–Wilk tests, parametric data were compared using the analysis of variance (ANOVA) followed by Tukey HSD tests. Nonparametric data were subjected to a Friedman's test followed by Tukey–Kramer tests.

## 3 Results

### 3.1 Antioxidative Activity of the Serum

**FIGURE 1:**
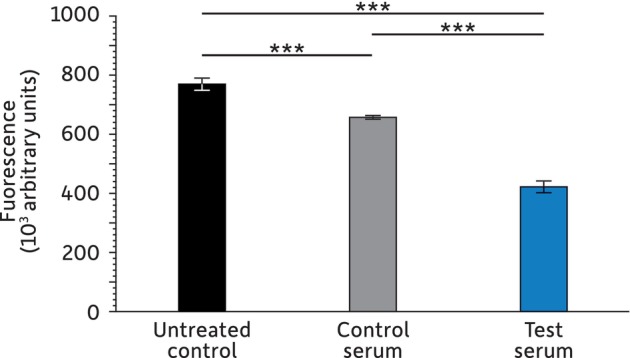
Quantification of ROS upon addition of hydrogen peroxide. Experiments were performed in triplicate for each condition with: ***p < 0.001.

As a first approach to evaluate the antioxidative properties of the test serum, we assessed its *in‐tubo* capacity to neutralize ROS upon addition of hydrogen peroxide (Figure 1).

The absence of hydrogen peroxide leads to no detectable ROS (data not shown), but its presence without serum corresponds to 100% ROS. The addition of the control serum leads to a significant 14.6% decrease in ROS (*p* < 0.001), while a reduction of 45.2% is measured with the test serum (*p* = < 0.001).

### 3.2 IL‐1α Release by Skin Tissue Models

UV radiation is a potent ROS inducer, and increased ROS levels are key intermediates of inflammation. To further assess the antioxidative soothing properties of the test serum, we evaluated its effect on UV‐induced IL‐1α release in EpiDerm^TM^ skin tissue models (Table 1).

**TABLE 1:** Quantification of IL‐1α release.

Treatment	IL‐1α (pg/mL)^a^
No serum—no UV	9.4 ± 1.1
Control serum + UV	148.6 ± 34.8
Test serum + UV	82.4 ± 19.2

^a^ Results are from triplicates of each condition.

Unprotected (no serum) and unstressed (no UV irradiation) EpiDerm^TM^ releases very low levels of a skin irritation biomarker such as IL‐1α. Conversely, those that received the control serum and were subjected to UV present an almost 16‐fold increase in the secretion of IL‐1α (*p* < 0.001). If instead of the control serum, the test serum is applied before UV irradiation, IL‐1α secretion is reduced by 44.6% (*p* = 0.028) but remains higher than for the unprotected‐unstressed control (*p* = 0.019).

### 3.3 HB‐EGF Assessment by Gene Expression

The Heparin‐Binding Epidermal Growth Factor (HB‐EGF) is an important marker of skin homeostasis. Results from RT‐qPCR quantification of its expression in skin explants (Table 2) indicate that both the control and the test serum significantly increase HB‐EGF expression compared to skin explants that received no serum.

**TABLE 2:** Relative quantification of HB‐EGF expression.

Treatment	Mean relative quantification (minimum—maximum)^a^	*p*‐value*
No serum	1.00 (0.50–2.01)	—
Control serum	15.51 (7.54–31.91)	0.010
Test serum	22.25 (14.08–35.73)	0.010

^a^ Results are from triplicates of each condition.

* *p*‐values are given versus skin explants that received no serum.

### 3.4 Elastin Fiber Length Assessment

**FIGURE 2:**
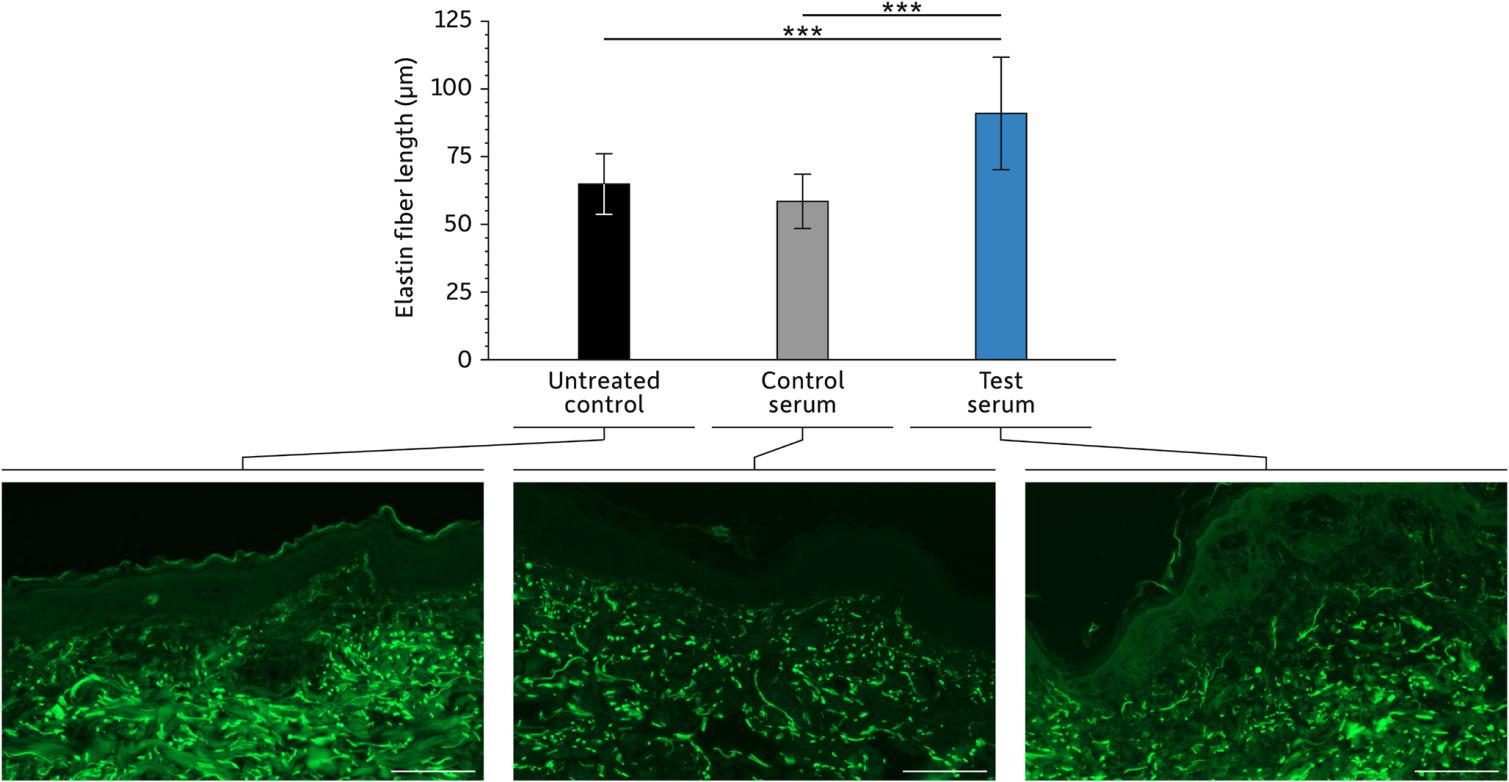
Elastin fiber length. Scale bar: 100 μm. For each treatment, 7–10 images were analyzed to calculate the length of three elastin fibers per image. For the statistical analysis: ***p < 0.001.

Another skin feature significantly affected by oxidative stress is the extracellular matrix. Consequently, we assessed the ability of the test serum to promote the synthesis of one of its components: elastin (Figure 2).

Compared to untreated explants, those that received topical applications of the control serum presented no difference in elastin fiber length (*p* = 0.304). Only explants treated with the test serum show a significant increase in elastin fiber length compared to untreated explants or control serum‐treated explants (*p* < 0.001 for both).

### 3.5 Instrumental and Clinical Evaluation

An important aspect of oxidative stress, especially when due to environmental stressors, is the visible changes it induces. Thus, subjects were recruited and the impact of twice‐daily application of the test serum for 28 days was evaluated.

Instrumental quantification of the Sa parameter, the mean skin surface roughness, shows no significant difference after 7 days of application (29.61 ± 1.16 at D0 versus 28.55 ± 0.98 at D7, *p* = 0.080). Still, a significant 6.0% decrease in Sa is observed after 28 days of application (27.84 ± 0.84, *p* = 0.002).

**FIGURE 3:**
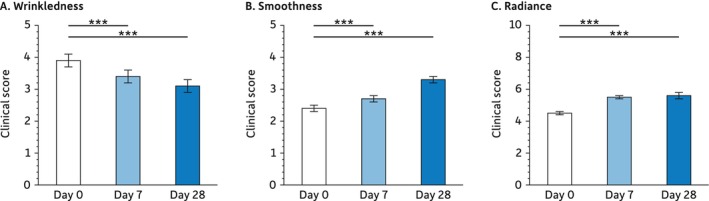
Clinical scoring of wrinkles, smoothness, and radiance. For the statistical analysis: ***p < 0.001.

This effect of the test serum on the skin surface is confirmed by the clinical scoring of skin wrinkles and smoothness (Figure 3). Expert scores indicate that both parameters are not only improved after 28 days (wrinkles: −20.5%, smoothness: +37.5%) but that this improvement is already significant after 7 days (wrinkles: −12.8%, smoothness: +12.5%). Besides, the test serum also improves skin radiance. Again, the effect is significant at both time points: Days 7 (+22.2%) and 28 (+24.4%).

The last point evaluated is the hydrating and protective properties of the test serum (Table 3). At all‐time points following application, skin hydration is improved, and transepidermal water loss (TEWL) is reduced. The effects of a single application are rapid, being already significant within 30 min and lasting for 8 hours. Repeated applications also lead to long‐term improvements.

**TABLE 3:** Instrumental measurements of the skin hydrating and protective properties of the test serum.

	Short‐term assessment^a^			Long‐term assessment^b^		
	T0	T 30 min	T 8 h	T0	Day 7	Day 28
Skin hydration (corneometry units)	35.2 ± 1.0	50.5 ± 2.0***	41.7 ± 1.3***	47.3 ± 1.4	54.8 ± 1.4***	56.7 ± 1.2***
Transepidermal water loss (g/h/m^2^)	11.7 ± 0.4	10.2 ± 0.3***	10.8 ± 0.4***	12.7 ± 0.4	12.1 ± 0.4**	11.6 ± 0.3***

*Note:* Statistical analysis is versus T0 with **p < 0.010 and ***p < 0.001.

^a^ Measurements on the volar side of a forearm.

^b^ Measurements at the level of the cheekbone.

### 3.6 Subjective Evaluation of the Serum

**FIGURE 4:**
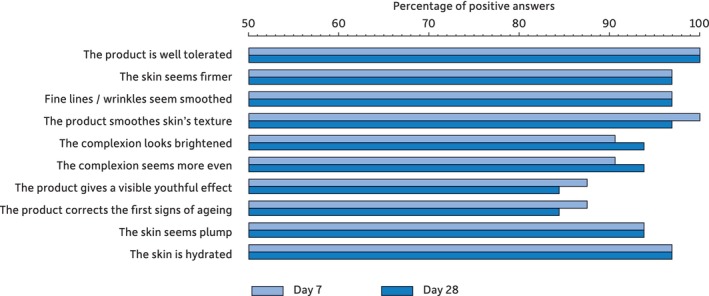
Percentage of “totally agree” or “mostly agree” positive answers in the self‐assessment questionnaire.

The subjects' perception of the effect of the test serum on various aspects of their facial skin revealed that it is well tolerated and that they rapidly perceived a positive impact (Figure 4).

## 4 Discussion

Having developed a new anti‐aging serum (test serum) that combines five antioxidants and hyaluronic acid, our goal was to assess its efficacy by comparing it to a serum devoid of antioxidants and hyaluronic acid (control serum). Results demonstrate that the test serum can inactivate ROS *in‐tubo*. Additionally, reduced IL‐1α release may result in decreased inflammation upon UV exposure. It also positively influences the extracellular matrix, as illustrated by the increased elastin fiber length. Furthermore, the control and the test serum both positively impact the expression of the heparin‐binding epidermal growth factor (HB‐EGF), an important marker of skin homeostasis.

These positive *in‐tubo*, in vitro, and ex vivo results led us to clinically evaluate the serum's anti‐aging properties. This study revealed that aging signs (wrinkles, smoothness, and radiance) evaluated by a dermatologist were significantly reduced within 7 days of twice‐daily applications. In addition, a single application of the test serum reduced TEWL and increased skin hydration within 30 min. These effects of a single application lasted up to 8 h, and repeated applications led to long‐term improvement. Finally, the test serum was very well tolerated and highly rated by the subjects.

In the absence of a control group, analyzes relied on comparison to baseline values. Therefore, we cannot state that the effects observed are due to the active ingredients. This is especially true for skin hydration, as the water‐based serum formulation itself could have had a positive impact.

Over the last decades, many anti‐aging compounds appeared: ceramide, retinol, hyaluronic acid, bakuchiol, etc. [9] Some are antioxidants, while others act through different mechanisms. Despite the wide range of antioxidants available, only a few are most commonly used, such as tocopherol and ascorbic acid [10], leaving many others with different characteristics and properties unexploited [9]. Combining these novel antioxidants to derive benefits from their individual and synergistic effects is of interest. Thus, we devised an anti‐aging serum by combining a unique blend of five antioxidants (tocopherol, ascorbic acid, niacinamide, astaxanthin, and ergothioneine) with hyaluronic acid.

Tocopherol and ascorbic acid have long‐proven efficacies. Both work in conjunction, creating a network of redox reactions staving off oxidative stress [11]. Tocopherol is a lipid‐soluble antioxidant that acts as a ROS scavenger, especially in cellular membranes [6, 11, 12, 13]. It acts by losing a proton, becoming a poorly reactive radical, which will regenerate into its original form. Upon topical application, it enters the stratum corneum upon topical application, prevents lipid peroxidation, minimizes photo‐damage, and reduces inflammation [11, 13]. Besides, tocopherol regulates the expression of several key genes, including the kinase C pathway, and downregulates the matrix metalloproteinase‐1 (MMP‐1), avoiding excessive collagen degradation [14, 15]. Ascorbyl glucoside is a water‐stable ascorbic acid derivative, which topical application results in photoprotection and improvement of aging signs [16, 17, 18]. It scavenges ROS and prevents lipid peroxidation by sequentially donating its electrons. It is also an essential cofactor in collagen synthesis—stimulating collagen I and III production and increasing fibroblast proliferation—and has versatile anti‐inflammatory properties. Finally, its topical application results in photoprotection and improvement of aging signs [17].

The three other antioxidants in the serum are less common but have interesting anti‐aging properties. Niacinamide is a nicotinamide adenine dinucleotide (NAD+) precursor. It contributes to energy metabolism by helping to restore the cellular NAD+ levels. It also has multiple antiaging effects, reduces oxidative stress and inflammation, improves the extracellular matrix, reinforces the skin barrier, and limits melanin synthesis [19]. All these effects would be achieved without specific molecular targets but by maintaining skin homeostasis through the regulation of the cellular redox status. Astaxanthin is a ketocarotenoid with multiple functions, potent antioxidant properties, and proven anti‐aging/anti‐inflammatory activities [20, 21]. It also improves skin hydration and elasticity, reduces pigmentation disorders, and limits UV‐induced DNA damage [19]. Finally, the last antioxidant in the serum is ergothioneine. It is a sulfur‐containing histidine derivative that accumulates in mitochondria, where it participates in the maintenance of normal ROS levels [22]. Besides mitigating aging signs, it has anti‐inflammatory and immunomodulatory activities, protects the skin barrier, and inhibits melanin synthesis [23].

Clinical evaluations of single antioxidants are limited, as is evidenced by the case of ascorbic acid [24]. Most studies evaluate cosmetic products, which Include several active ingredients. These products also differ in their chassis composition and evaluation methods. Thus, as is the case in our study, determining and quantifying the very impact of every component is almost impossible. Although, the anti‐aging properties of antioxidants are well recognized, relating our work to previous studies is challenging. Thus, demonstrating the superiority of the antioxidant blend in the serum will require further investigation.

Despite this limitation, the observed activities of the anti‐oxidants in the serum composition explain the results obtained. Furthermore, the serum contains hyaluronic acid, which has water retention capacities, improving very rapidly skin hydration and reducing aging signs, including wrinkle severity, after a few weeks [24]. This likely contributes to the effects evidenced. Yet, determining the exact role of each of these compounds is far beyond the scope of this work.

## 5 Conclusions

Combining antioxidants in an anti‐aging cosmetic is not a novelty. The rationale is to take advantage of their different distribution within the skin and its cells, their complementary activities, and to benefit from synergetic effects that reinforce their overall efficacy. We went one step further, by using a unique blend of five antioxidants combined with hyaluronic acid. The resulting combination has antioxidative and anti‐inflammatory properties, promoting skin homeostasis and elastin fiber length in vitro. Upon clinical evaluation, the cosmetic serum demonstrated improvement of wrinkles, skin smoothness, and radiance, while also increasing skin hydration and protecting the skin barrier. These effects are explained by the serum's composition, especially the blend of antioxidants and hyaluronic acid. Yet, assessing the serum's long‐term effects would be of interest to fully explore its benefits, particularly on features requiring a longer period to show improvement, such as skin color homogeneity and skin physical characteristics resulting from extracellular matrix enhancement.

## Author Contributions

**Sayantani Goswami** and **Jin Namkoong:** conceptualization. **Sayantani Goswami**, **Jin Namkoong**, **Marwa El Hajoui,** and **Joanna Wu:** methodology. **Sayantani Goswami** and **Jin Namkoong:** formal analysis. **Ewelina Lesniak** and **Joanna Wu:** resources. **Sayantani Goswami** and **Jin Namkoong:** data curation. **Sayantani Goswami** and **Jin Namkoong:** writing – original draft preparation. **Sayantani Goswami**, **Jin Namkoong**, **Marwa El Hajoui**, **Joanna Wu,** and **Ewelina Lesniak:** writing – review and editing. **Joanna Wu** and **Ewelina Lesniak:** supervision. **Joanna Wu** and **Ewelina Lesniak:** project administration. All authors have read and agreed to the published version of the manuscript.

## Ethics Statement

The instrumental and clinical evaluation of the cosmetic serum is a noninvasive cosmetic evaluation. Performed in Italy under the supervision of Colgate‐Palmolive Company (USA), this study was approved by the U.S. Investigational Review Board Inc. under the reference of U.S. IRB2022CP/09. It strictly conformed to the principles of the Declaration of Helsinki and followed Good Clinical Practices.

## Conflicts of Interest

All authors are full‐time employees of Colgate‐Palmolive Company.

## Supporting information


Supporting Information S1.

## Data Availability

Data are available upon reasonable request to the corresponding author.
