# Supplementary material for: In Vitro, Ex Vivo, Instrumental, and Clinical Assessment of a Novel Anti‐aging Serum Targeting Oxidative Stress
Source: J Cosmet Dermatol. 2025 Apr 3;24(4):e16664. doi: 10.1111/jocd.16664 (PMC11967377; doi:10.1111/jocd.16664)
Supplement: Supplementary file 1 — Supporting Information S1. [file JOCD-24-e16664-s001.docx]

**Detailed INCI composition of the test and control serum**

**Test Serum Ingredient List:**

Water, Glycereth-26, Methyl Propanediol, Niacinamide, Ascorbyl Glucoside, PPG-26-Buteth-26, PEG-40 Hydrogenated Castor Oil, Pentylene Glycol, Tocopherol, Caprylyl Glycol, Hydrolyzed Hyaluronic Acid, Mannitol, Sodium Citrate, Xanthan Gum, Fragrance, Sodium Benzoate, Citric acid, Sodium Hydroxide, Sodium Phytate, Adenosine, Sucrose Palmitate, Glyceryl Linoleate, *Prunus Amygdalus Dulcis* (sweet almond oil), Sodium Chloride, *Simmondsia Chinensis* (Jojoba) seed oil, *Haematococcus pluvialis* extract, Ergothioneine, Glucose, Sodium Hyaluronate, Potassium Chloride, Potassium Sorbate, Calcium Chloride, Magnesium Sulphate, Glutamine, Sodium Phosphate, Ascorbic Acid, Sodium Acetate, Lysine HCl, Arginine HCl, Alanine, Histidine HCl, Valine, Leucine, Threonine, Isoleucine, Tryptophan, Phenylalanine, Tyrosine, Glycine, Polysorbate 80, Serine, Cystine Cyanocobalamine, Glutathione, Asparagine, Aspartic acid, Ornithine HCl, *Helianthus Annuus* (Sunflower) seed oil, Glutamic Acid, Nicotinamide Adenine Dinucleotide, Proline, Methionine, Taurine, Hydroxyproline, Glucosamine HCl, Coenzyme A, Sodium Gluconate Thiamine Diphosphate, Retinyl Acetate, Inositol, Niacin, Pyridoxine HCl, Biotin, Calcium Pantothenate, Riboflavin, Sodium Tocopheryl Phosphate, Thiamine HCl, Folic Acid.

**Control Serum Ingredient List:**

Water, Glycereth-26, Methyl Propanediol, PPG-26-Buteth-26, PEG-40 Hydrogenated Castor Oil, Pentylene Glycol, Caprylyl Glycol, Mannitol, Sodium Citrate, Xanthan Gum, Fragrance, Sodium Benzoate, Citric acid, Sodium Hydroxide, Sodium Phytate, Potassium Chloride, Potassium Sorbate, Calcium Chloride, Magnesium Sulphate, Glutamine, Sodium Phosphate, Sodium Acetate, Polysorbate 80.
